# Supplementary material for: Reconciling Mining with the Conservation of Cave Biodiversity: A Quantitative Baseline to Help Establish Conservation Priorities
Source: PLoS One. 2016 Dec 20;11(12):e0168348. doi: 10.1371/journal.pone.0168348 (PMC5173368; doi:10.1371/journal.pone.0168348)
Supplement: S1 Dataset — (ZIP) [file pone.0168348.s002.zip › Taxa/Serra Norte/SN_2013/N4E/Lista N4E-46.pdf]

CAVIDADE N4E-0046

Classe	Ordem	Fam/Outros	Gên/Outros	Espécie	S-U	U-S	Zon.
Annelida	Oligochaeta			jovem		2	P
Arachnida	Acari	(Parasitengonina)		sp.4		X	P
Arachnida	Acari	Neothyridae	*Diplothyrus*	sp.1	X		P
Arachnida	Acari	Rhagidiidae		sp.4		X	P
Arachnida	Araneae	Araneidae		jovem	X		P
Arachnida	Araneae	Ctenidae	*Enoploctenus*	sp.1		1	E
Arachnida	Araneae	Ctenidae		jovem		2	P
Arachnida	Araneae	Drymusidae		jovem		X	P
Arachnida	Araneae	Filistatidae		jovem	X		P
Arachnida	Araneae	Ochyroceratidae	*Ochyrocera*	sp.1		X	P
Arachnida	Araneae	Ochyroceratidae	*Ochyrocera*	sp.2	X		P
Arachnida	Araneae	Pholcidae	*Leptopholcus*	sp.1		X	P
Arachnida	Araneae	Pholcidae	*Mesabolivar*	sp.1	X	X	P
Arachnida	Araneae	Pholcidae	Ninetinae	sp.1	X		P
Arachnida	Araneae	Salticidae		sp.6	X		P
Arachnida	Araneae	Theridiosomatidae	*Plato*	sp.1		X	P
Arachnida	Pseudoscorpiones	Olpiidae		sp.1	X		P
Diplopoda	Polydesmida	Fuhrmannodesmidae		sp.1		X	P
Diplopoda	Polydesmida	Pyrgodesmidae		sp.1		X	P
Entognatha	Collembola	Cyphoderidae		sp.1		X	P
Entognatha	Collembola	Entomobryidae		sp.1	X		P
Entognatha	Collembola	Entomobryidae		sp.3	X		P
Entognatha	Diplura	Campodeidae		sp.1	X		P
Insecta	Blattodea			jovem	1		P
Insecta	Coleoptera	Chrysomelidae		sp.14	X	X	P
Insecta	Diptera	Cecidomyiidae	Cecidomyiinae	sp.	X	X	P
Insecta	Diptera	Psychodidae	Phlebotominae	sp.	X	X	P
Insecta	Diptera	Tipulidae	Tipulinae	sp.		X	P
Insecta	Heteroptera	Lygaeidae		jovem		X	P
Insecta	Homoptera	Cixiidae		sp.3	X	X	P
Insecta	Hymenoptera	Formicidae	*Camponotus*	sp.2	X		P
Insecta	Hymenoptera	Formicidae	*Dolichoderus*	aff. *bispinosus*		X	P
Insecta	Hymenoptera	Formicidae	*Hypoponera*	sp.2		X	P
Insecta	Hymenoptera	Formicidae	*Pachycondyla*	sp.2	X	X	P
Insecta	Hymenoptera	Formicidae	*Pheidole*	sp.2	X	X	P
Insecta	Lepidoptera	Gelechioidea		sp.	X		P
Insecta	Lepidoptera	Noctuoidea		sp.5		1	P
Insecta	Neuroptera	Myrmeleontidae		jovem		1	P
Insecta	Orthoptera	Gryllidae	Nemobiinae	jovem		1	P
Insecta	Orthoptera	Phalangopsidae	*Paraclodes*	sp.	10	1	E/P
Insecta	Psocoptera			jovem		X	P
Insecta	Thysanura	Nicoletiidae		sp.1	X		P
Gastropoda	Pulmonata	Systrophiidae	*Happia*	sp.		X	P
Malacostraca	Isopoda	Dubioniscidae		sp.1	X		P
Malacostraca	Isopoda	Philosciidae		sp.2		X	P
Turbellaria	Tricladida			sp.2		2	P
Amphibia	Anura	Strabomantidae	*Pristimantis*	cf. *fenestratus*	1		P
Mammalia	Chiroptera	Emballonuridae	*Peropteryx*	*kappleri*	3	1	E
Reptilia	Squamata	Gekkonidae	*Thecadactylus*	sp.		1	E
